# Survival analysis tools in genomics research

**DOI:** 10.1186/s40246-014-0021-z

**Published:** 2014-11-25

**Authors:** Xintong Chen, Xiaochen Sun, Yujin Hoshida

**Affiliations:** Liver Cancer Program, Tisch Cancer Institute, Division of Liver Diseases, Department of Medicine, Icahn School of Medicine at Mount Sinai, 1470 Madison Avenue, Box 1123, New York, NY 10029 USA

**Keywords:** Survival analysis, Software, Web application, Genomic database

## Abstract

There is an increasing demand to determine the clinical implication of experimental findings in molecular biomedical research. Survival (or failure time) analysis methodologies have been adapted to the analysis of genomics data to link molecular information with clinical outcomes of interest. Genome-wide molecular profiles have served as sources for discovery of predictive/prognostic biomarkers as well as therapeutic targets in the past decade. In this review, we overview currently available software, web applications, and databases specifically developed for survival analysis in genomics research and discuss issues in assessing clinical utility of molecular features derived from genomic profiling.

## Survival analysis in genomics research

With the increasing capability to perform genome-wide molecular characterization of clinical specimens, making clinical implication of genomic aberrations has become a more relevant topic. The decreasing cost of the assays has facilitated accumulation of genomic profiles of sizable clinical cohorts, with which more reliable molecular prognostic analysis has become possible. Also, expanding clinical contexts covered by the studies/datasets has enabled exploration of clinically more relevant predictive/prognostic biomarkers from genomic data [1]. Here, the major interest is the association of genomic features with clinical outcomes, including response to certain treatment and prognosis of the patients under specific clinical scenarios.

Clinical outcome especially prognosis is often presented as the time period between the start and end of the clinical observation in combination with a binary status information, indicating whether or not each patient had a clinical event of interest, e.g., death, cancer recurrence, and drug response. In contrast to laboratory experiment-derived data, clinical outcome data are generally incomplete because of the missing observation of the clinical event. For example, in the case of analyzing time to cancer recurrence after surgery, some patients who are still recurrence free during the study period may develop recurrence later, i.e., it is uncertain whether the patient should be classified into recurrence-positive or recurrence-negative group. Such situation, where a true outcome is still unknown, is treated as a censored observation, and the observation time is incorporated in the analysis. This type of analysis is called “survival” or “failure time” analysis, for which various biostatistical analysis methodologies are already available. These methodologies have been adapted for the analysis of genomic datasets with modifications to accommodate the high-dimensional data structure by utilizing correction methods for highly multiple hypothesis testing [2].

The accumulated genomic datasets with clinical outcome information have led to a new paradigm of biomarker research, i.e., *in silico* discovery and/or validation of predictive/prognostic molecular biomarkers. In this article, we overview currently available software, web applications, and databases specifically developed for integrative analysis of survival and genomic data. We also discuss current limitations mostly residing on the clinical study design side and how we could methodologically overcome these challenges to facilitate the development of molecular biomarkers with clinical utility.

## Tools and resources for survival analysis in genomics research

The major tasks of survival analysis in genomics research include 1) survey/identify genomic feature(s) correlated with survival data and 2) evaluate/validate survival data correlation for predefined genomic feature(s). There are several freely available tools to complete the tasks for users with a wide range of informatics capability and fluency (Table 1). Significance Analysis of Microarrays (SAM) is one of the earliest software to identify genomic feature(s) correlated with biological and/or clinical phenotypes of interest, including time-to-event clinical outcome by using Cox score [3,4]. A similar algorithm is implemented as modules of the GenePattern software, a generic genomic data analysis environment and toolkit [5]. GenePattern LoocvSurvival module enables generation of a robust prognostic gene signature based on leave-one-out cross-validation scheme [6]. Cox regression-based method together with time-dependent receiver operating characteristic (ROC) curve analysis was also reported [7]. Net-Cox is a method based on Cox regression modeling using the information of co-regulated multiple genes, which was reported to improve replication of the prognostic model [8]. survcomp is an R-based Bioconductor [9] package for survival risk model comparison based on time-dependent ROC curve and c index [10].

**Table 1:** **Software for genomic feature-based survival analysis**

**Software**	**User interface (programming language)**	**Functionality**	**Reference**	**URL**
Significance Analysis of Microarrays (SAM)	Graphical (Excel add-on), command-line (R)	Feature selection	[3,4]	[11]
GenePattern^a^	Graphical	Feature selection, assessment of survival association, model building	[5]	[12,13]
Partial Cox regression analysis	Command-line (R)	Feature selection, assessment of survival association, model building	[7]	^b^
Net-Cox	Command-line (Matlab)	Feature selection, assessment of survival association, model building	[8]	[14]
survcomp	Command-line (R)	Model comparison	[10]	[15]

^a^SurvivalGene, PrognosticGene, and LoocvSurvival modules deposited in [13].

^b^Source code available upon request to the authors.

The ever-expanding repositories of genomic datasets with clinical outcome information have been serving as resources to build web-based tools/resources for survival-related genomic analysis (Table 2). NCBI Gene Expression Omnibus (GEO) [16] and EBI ArrayExpress [17] are generic databases of a variety of genomic datasets with or without clinical outcome information. The Cancer Genome Atlas (TCGA) is a multi-institutional project generating a wide range of genomic data, which are made publicly available together with rich clinical annotations including outcome data [18]. Several survival analysis-focused web applications have also been built based on these resources. Oncomine is an intensively curated genomics database with a special focus on oncology research, providing functionalities of survival-related analysis for datasets with relevant sample annotations [19]. cBioPortal is a web-based resource that enables graphical user interface (GUI)-based intuitive interrogation of a wide range of omics datasets from TCGA and Cancer Cell Line Encyclopedia (CCLE) [20] datasets and, when available, survival data analysis including Kaplan-Meier curve and log-rank test [21]. Similar web-based resources combining genomic/clinical database and analysis tools that enable single/multiple gene-based prognostic assessment include Kaplan-Meier Plotter [22], PrognoScan [23], GOBO [24], Recurrence Online [25], PROGgene [26], bc-GenExMiner [27], ITTACA [28], SurvExpress [29], and G-DOC Plus [30]. These resources assembled publicly or privately available datasets from GEO, ArrayExpress, TCGA, and/or private solicitation/deposition and enable survival analysis based on prefixed or user-defined cutoff for prognostic subgrouping of the patients. Some of them support subgroup analysis and/or multivariable analysis with clinical prognostic variables when available. Some support survival classifier based on multiple genes (or gene signature) using preset algorithms such as averaging or multivariable Cox regression modeling. Breast Cancer Competition (BCC) is a collection of tools to facilitate collaborative genomic classifier building and testing, which was recently used to develop breast cancer prognostic models based on competition between multiple data analysis groups [31]. These tools are readily available to analyze user’s own genes or survival models in a variety of diseases, tissue types, and clinical contexts when available.

**Table 2:** **Web applications with database for genomic feature-based survival analysis**

**Web application/database**	**Analyzable genetic feature**	**Covered diseases**	**Reference**	**URL**
Oncomine	Multiple	Cancer	[19]	[32]
cBioPortal	Multiple	Cancer (37 types)	[21]	[33]
Kaplan-Meier Plotter	Single	Cancer (breast, ovarian, lung)	[22]	[34]
PrognoScan	Single	Cancer (14 types)	[23]	[35]
GOBO	Multiple	Cancer (breast)	[24]	[36]
Recurrence online	Multiple	Cancer (breast)	[25]	[37]
PROGgene	Single/multiple	Cancer (21 types)	[26]	[38]
bc-GenExMiner	Single	Cancer (breast)	[27]	[39]
ITTACA	Single	Cancer (7 types)	[28]	[40]
SurvExpress	Multiple	Cancer (20 types)	[29]	[41]
G-DOC plus	Multiple	Cancer (9 types), non-cancer (3 types)	[30]	[42]

Accessed in October 2014.

## Toward genome-based biomarkers with real clinical utility

*In silico* biomarker validation could be a substantially more cost-effective strategy for biomarker development, which typically requires costly and lengthy processes. Despite the exponentially expanding genomic databases and associated survival analysis tools and resources, clinically deployed genome-based biomarkers are still scarce, highlighting the unresolved challenges in biomarker development from genomic studies [43]. One major issue is the clinical study design, which derives the genomic dataset. Predictive/prognostic biomarkers must follow predefined specific study plan to demonstrate their validity and clinical utility. In general, such biomarkers and models should be clearly defined and independently evaluated in prospectively enrolled patients. The guidelines for assessment of prognostic marker (REMARK) [44], diagnostic marker (STROBE) [45], and cohort study (STARD) [46] are available to ensure the quality and validity of the biomarkers. However, a vast majority of available genomic datasets rarely meet these requirements because they were generated by using samples of convenience, i.e., biospecimens readily available to the researchers, which were retrospectively collected without predetermined intention of biomarker development or assessment. That is, prognostic genes identified through analysis of the databases may not or less likely to be clinically reliable or reproducible as biomarkers. Quality grading for the study design in the genomic databases such as the one proposed by Simon and colleagues, A (prospective study), B (retrospective analysis of previous prospective study samples), C (prospective/observational), and D (retrospective/observational) [47], will help speculate the reliability of the survival analysis result yielded from each specific dataset. Generation of future genomic data with special attention on these study design-related issues will enable highly reliable computational validation of new biomarkers.

Obviously, the primary goal of this type of exploratory analysis is to determine or speculate clinical outcome association of genomic features. However, if the features selected through the surveillance are further considered as candidates for clinical diagnostic development, there is another issue that needs to be considered. Clinical decision making is generally made according to well-defined, specific clinical contexts that are often summarized in a diagram or flow chart in the clinical practice guidelines. For a molecular biomarker to be considered as a clinical test to support the system of clinical decision making, the marker must demonstrate clinically meaningful utility in terms of magnitude of benefit, feasibility of clinical implementation, and cost in association with the system of existing clinical decision making system/algorithm. It will be technically feasible to incorporate such clinical framework in the aforementioned web-based tools of genomic survival analysis by engaging disease domain experts in their development.

Clinically applicable molecular biomarkers must yield reproducible and robust measurements in real-world clinical setting with clinically acceptable logistical complexity and cost to justify their use. The lack of reproducibility of the measurement especially for transcript-based biomarkers has been the major technical obstacle in clinical deployment of genome-based biomarkers [48]. Recent development of digital biomolecule counting technologies without target amplification has been overcoming this challenge by enabling a more sensitive and robust measurement of a variety of analytes, including DNA, RNA, and protein, as well as chemical modifications of these molecules [49]. Assay technologies that are specifically designed to generate genomic data from real-world clinical specimens, e.g., formalin-fixed paraffin-embedded tissues, will further expand the informatics resources with rich clinical contexts/scenarios and enhance our capability of *in silico* biomarker research. To accommodate requirements from the regulatory agencies for biomarkers such as FDA in the web-based resources may also help facilitate biomarker development. Two additional challenges in bringing genome-based prognostic biomarkers into clinics are reimbursement for the assays from health insurance companies and education of patients and physicians. To make the web-based genomic survival analysis resources accessible to broader communities outside of biomedical research by integrating them with clinical decision support system (CDSS) in electronic health record (EHR) may help resolve these issues and eventually facilitate clinical translation of genome-based prognostic biomarkers.

## Abbreviations

FDR: False discovery rate
ROC: Receiver operating characteristic
